# The *HTS barcode checker* pipeline, a tool for automated detection of illegally traded species from high-throughput sequencing data

**DOI:** 10.1186/1471-2105-15-44

**Published:** 2014-02-06

**Authors:** Youri Lammers, Tamara Peelen, Rutger A Vos, Barbara Gravendeel

**Affiliations:** 1 Naturalis Biodiversity Center, Darwinweg 4, 2333 CR Leiden, The Netherlands; 2 Dutch Customs Laboratory, Kingsfordweg 1, 1043 GN, Amsterdam, The Netherlands; 3 University of Applied Sciences Leiden, Zernikedreef 11, 2333 CK, Leiden, The Netherlands; 4 Leiden University, Faculty of Science, Einsteinweg 55, 2333 CC, Leiden, The Netherlands

**Keywords:** Biodiversity informatics, High-throughput sequencing, Taxonomy, Wildlife forensics

## Abstract

**Background:**

Mixtures of internationally traded organic substances can contain parts of species protected by the Convention on International Trade in Endangered Species of Wild Fauna and Flora (CITES). These mixtures often raise the suspicion of border control and customs offices, which can lead to confiscation, for example in the case of Traditional Chinese medicines (TCMs). High-throughput sequencing of DNA barcoding markers obtained from such samples provides insight into species constituents of mixtures, but manual cross-referencing of results against the CITES appendices is labor intensive. Matching DNA barcodes against NCBI GenBank using BLAST may yield misleading results both as false positives, due to incorrectly annotated sequences, and false negatives, due to spurious taxonomic re-assignment. Incongruence between the taxonomies of CITES and NCBI GenBank can result in erroneous estimates of illegal trade.

**Results:**

The *HTS barcode checker* pipeline is an application for automated processing of sets of 'next generation’ barcode sequences to determine whether these contain DNA barcodes obtained from species listed on the CITES appendices. This analytical pipeline builds upon and extends existing open-source applications for BLAST matching against the NCBI GenBank reference database and for taxonomic name reconciliation. In a single operation, reads are converted into taxonomic identifications matched with names on the CITES appendices. By inclusion of a blacklist and additional names databases, the *HTS barcode checker* pipeline prevents false positives and resolves taxonomic heterogeneity.

**Conclusions:**

The *HTS barcode checker* pipeline can detect and correctly identify DNA barcodes of CITES-protected species from reads obtained from TCM samples in just a few minutes. The pipeline facilitates and improves molecular monitoring of trade in endangered species, and can aid in safeguarding these species from extinction in the wild. The *HTS barcode checker* pipeline is available at https://github.com/naturalis/HTS-barcode-checker.

## Background

The Convention on International Trade in Endangered Species of Wild Fauna and Flora (CITES) entered into force in 1975, and aims to control and regulate trade in endangered species. The convention produces appendices that list taxa (at various taxonomic levels, e.g. species, genera, families) in which trade is controlled or prohibited. These appendices are agreed upon and amended at the Conference of Parties meetings and are available online [1]. There are three appendices: Appendix I includes taxa threatened with extinction; Appendix II contains taxa not threatened with immediate extinction but for which regulation is required to avoid overexploitation that might threaten survival in the wild; Appendix III includes taxa for which one CITES member country requested the involved CITES parties for assistance in controlling the international trade.

CITES appendix-listed taxa cannot be traded internationally without permits from the exporting and importing country. Monitoring of trade in CITES taxa is a challenge to customs authorities worldwide and is especially difficult when parts such as antlers, horns, leaves, roots, or powders are used in mixtures such as in Traditional Chinese Medicines (TCMs). During manufacturing of TCMs, parts are often processed (ground, heated, dried, blended with other products), and this makes taxonomic identification based on chemical, chromatographic or morphological methods challenging [2,3]. Labels often do not provide sufficient warranties about the actual contents of a product, because listed ingredients may be absent whereas others may be included. New methods are therefore needed to protect both consumers and producers from fraud, and endangered species from overexploitation.

DNA barcoding [4] is a powerful new tool in the emerging field of wildlife forensics since species composition of mixtures can be reconstructed by sequencing and identifying variable barcoding markers. The mitochondrial marker *COI* is frequently used for animals; for plants, the official Barcode of Life (BoLD) plastid markers *matK* and *rbcL* are used in addition to the nuclear ribosomal Internal Transcribed Spacer (nrITS). DNA barcodes can be identified by querying them against databases such as NCBI GenBank or BoLD [5], to which reference sequences of animal and plant species have been submitted over the past 10 years. (The latter database includes images of reference specimens and additional sampling details in addition to sequence data). The availability of increasingly complete reference databases has opened up the way for DNA barcoding to become a standard tool for regulatory institutions worldwide to control illegal trade in endangered species.

High-throughput sequencing (HTS) techniques, e.g. those based on platforms such as Illumina HiSeq, IonTorrent, PacBio and Roche 454 [6,7], yield increasingly large volumes of barcode sequences. This leads to a greater identifying potential for complex species samples such as TCMs, at low cost. The process of going through a set of identified sequences and manually comparing them to the CITES appendices is labor intensive and error prone for several reasons. Firstly, HTS continues to increase the volume of reads, which in turn increases the time to process the data. Secondly, the CITES appendices are only available as HTML documents on the internet, which makes manual verification of sequencing results against the appendix-listed taxa labor intensive. Thirdly, the CITES appendices often list higher taxa (e.g. genera, families) whereas reference sequences are annotated to species level, requiring the correct expansion of the CITES taxa to the level to which sequences are annotated. Fourthly, false positive hits can occur for DNA barcodes deposited in NCBI GenBank, as it occasionally has incorrect taxonomic name annotations. Lastly, taxonomies of the CITES appendices and NCBI GenBank are not always congruent, and this can lead to erroneous conclusions about illegal trade in endangered species.

To address these challenges we have developed an open-source, freely available pipeline that automates the identification and CITES listing verification steps to enable efficient scanning of large sample sequence datasets, and allows for quick detection of presence of DNA barcodes derived from protected species.

## Implementation

### Overview

**Figure 1:**
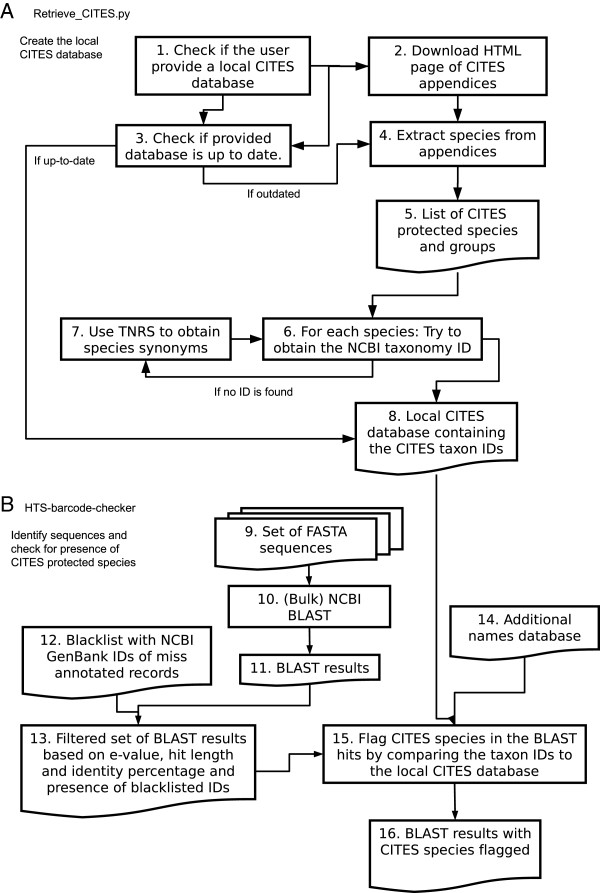
**Steps of the analysis pipeline.** Section **A** shows the process of both controlling the version of the local CITES database as well as updating the database with NCBI taxonomy IDs of CITES-protected taxa. Section **B** shows the process of running a BLAST search on the user input FASTA file, filtering the output according to minimum BLAST quality settings and blacklisted GenBank entries and flagging CITES protected taxa by comparing the BLAST hits against the local CITES database (and optionally other databases).

The *HTS barcode checker* pipeline verifies whether a sequence originates from a CITES-protected taxon by comparing it with data in NCBI GenBank [8] using BLAST algorithms [9]. We chose to use BLAST over other similarity search tools as this exposes the annotated NCBI GenBank database as reference material (both via online and standalone searching). Since we rely on NCBI taxonomic identifiers (taxon IDs) to decide whether a BLAST hit is from a CITES-protected taxon this is a requirement for the HTS barcode checker pipeline. The taxon IDs of the resulting BLAST hits are subsequently compared to a list of taxon IDs that correspond to CITES-listed taxa. Any putative matches are reported back to the user including the immediately surrounding context of the CITES appendix text. The steps of the pipeline are shown in Figure 1 and are explained in more detail below.

The *HTS barcode checker* is written in python and uses the *biopython*[10], *beautiful-soup*[11] and *requests*[12] packages to handle FASTA sequences and communicate with the various APIs and web services used, such as NCBI GenBank and the PhyloTastic TNRS service. The steps of the pipeline are shown in Figure 1 and are explained in more detail below.

### Local names databases

As an offline process, a local database is maintained containing the NCBI taxon IDs of CITES-listed taxa. The distribution contains a ready-to-use copy of this database; at the time of writing this copy is based on the CITES appendices of June 12^th^ 2013.

By default the *HTS barcode checker* will compare the CITES database to the latest version of the online CITES appendices during steps that are automatically carried out by the Retrieve_CITES.py script (Figure 1 – step 1-3, section A). If the local version is out of date a new version will be created by scanning the CITES appendices and retrieving the names of CITES-protected species and the appendix numbers in which they occur (Figure 1 – step 4-5). For each entry in the CITES appendix the corresponding taxon ID is initially retrieved using approximate string searches in the NCBI taxonomic database (Figure 1 - step 6). Since an entire genus or family can be listed in the CITES appendix (for example: *Dendrobium* or Orchidaceae), higher taxa are expanded into the lower, terminal ranks to which GenBank sequences are annotated. When no taxon ID can be obtained, a taxonomic name resolution web service (TNRS) [13,14] is used (Figure 1 - step 7) to obtain a list of synonyms, based on which the pipeline retries to obtain and expand taxon IDs. During testing, synonyms were successfully retrieved for the CITES-protected plant species *Euphorbia capsaintemariensis* (syn. *E. cap-saintemariensis*), *Laelia jongheana* (syn. *Cattleya jongheana*), *Crysaldiocarpus decipiens* (syn. *Dypsis decipiens*), *Sarracenia rubra* ssp. *jonesii* (syn. *S. jonesii*), and *Platymiscium pleiostachyum* (syn. *P. parviflorum*). The resolved, species-level NCBI taxon IDs for CITES-protected taxa are locally stored along with CITES appendix information and NCBI taxon names in a comma-separated values (CSV) file that can be read with standard spreadsheet software.

Additional such databases containing taxon IDs of species that are not listed on the CITES appendices but might still be of interest can be provided by the user. For example, users can provide a database of NCBI taxon IDs of controversial species that may be considered synonymous to CITES-protected species. An example of this is given in Table 1.

**Table 1:** User-specified, additional names database

**Taxon ID**	**Taxon**
44587	*Panax omeiensis* J. Wen, ined.
44686	*Panax sinensis* J. Wen, ined.

Examples of species of *Panax* that are listed in NCBI GenBank as distinct species, but are considered to be synonyms of *P. ginseng* (listed on CITES Appendix 2) by most other taxonomic databases, including the CITES Appendices, due to their unpublished status and close genetic similarity to *P. ginseng*.

### Sequence identification

To identify putative CITES-listed taxa from DNA barcode sequence data, the HTS-barcode-checker script (Figure 1 – section B) takes a set of sequences in FASTA format and searches these either using BLAST against the NCBI GenBank database (Figure 1 - step 4) or a local BLAST search with the NCBI-BLAST + tool [15] (Figure 1 – step 9-11). All GenBank databases and BLAST algorithms are supported; by default the nucleotide database *nr* is searched using the *blastn* algorithm.

It is recommended that large NGS sets (thousands to millions of reads) are first clustered into Operational Clustered Taxonomic Units (OCTUs) with tools such as CD-HIT [16]. CD-HIT is a relatively fast cluster program that clusters sequences based on sequence similarity. Sequences are sorted based on decreasing sequence length, with the first sequence acting as a cluster seed. All subsequent sequences are matched against the seed, depending on the similarity threshold they are placed in the same cluster or serve as a new cluster seed.

By clustering the sequences redundancy is reduced, which will decrease BLAST time. For example a sample containing 79,000 IonTorrent reads could be clustered to a set of of 3,845 representative sequences at 97% sequence similarity. Overloading of the NCBI servers is prevented by a default timeout between BLAST submits, however for larger datasets it is recommended to use a local BLAST database in combination with the NCBI-BLAST + tool (obtainable from [17]). The update_blastdb.pl tool that comes with the NCBI-BLAST + package can be used to download and maintain local copies of the GenBank databases (18GB for the nucleotide dataset).

By default the pipeline filters BLAST results in a multistep process. Firstly, low quality hits are discarded. By default these are hits with e-values larger than 0.05 to avoid potential false positive results, hits shorter than 100bp that may not contain the sequence diversity needed to accurately determine the species [18], and/or hits with a lower sequence identity than 97% which is generally used to determine sequences to species level [19,20], though the user can alter these settings if needed, for example when dealing with shorter Illumina fragments. Secondly, blacklisted NCBI GenBank accessions are filtered out (Figure 1 – step 12). Since NCBI GenBank contains erroneous taxonomic names [21], the pipeline uses a user-editable blacklist of NCBI GenBank accession numbers for which taxonomic identification is known to be incorrect. An example of this is given in Table 2. For each hit that passes both BLAST quality and blacklist filtering (Figure 1 - step 13) the taxon ID is obtained from the sequence record. This taxon ID is then matched against the local CITES database and any additional user-provided taxon ID databases (Figure 1 - step 14-15) to determine if the sequence originates from a CITES-protected taxon.

**Table 2:** User-specified blacklist

**GenBank accession**	**Current (erroneous) annotation**	**Correction**
EF090607	*Gastrodia elata*	Nyctaginaceae
EU135905	*Gastrodia elata*	Nyctaginaceae

Examples of NCBI GenBank accessions placed in our user-specified blacklist, erroneously listed on GenBank as belonging to *Gastrodia elata*, a highly endangered orchid (monocot) species listed on CITES appendix I, but containing nrITS sequences of the not endangered eudicot Nyctaginaceae instead.

### Output

The final result is a tab-separated values file (TSV) containing the query sequences, BLAST hits and, in case a CITES-listed taxon was found, also the surrounding textual context from the relevant appendix. A condensed example of the output is shown in Table 3. CITES appendices contain multiple exceptions for certain taxa, e.g. based on their geographic location, domestication status or the enforcement of trade quota. The pipeline is unable to respond to these exceptions as they are not made available in a structured format. Therefore, all results that match the names listed in the CITES appendices are flagged and all surrounding context is reported to the user.

**Table 3:** Summarized test cases

**Sample**	**Query**	**Identification (% identity)**	**CITES taxon**	**Taxon ID**	**Appendix**
Incense cone	Cluster 0	*Aquilaria sinensis* (98.76%)	*Aquilaria* spp.	210372	2
Agarwood chips	Cluster 22	*A. rugosa* (98.35%)	*Aquilaria* spp.	314115	2
*Dendrobium* stem	Cluster 5	*Dendrobium cariniferum* (98.2%)	Orchidaceae spp.	179352	2
*Dendrobium* stem	Cluster 1400	*D. dixanthum* (97.63%)	Orchidaceae spp.	335151	2
*Dendrobium* stem	Cluster 4500	*D. williamsonii* (99.18%)	Orchidaceae spp.	161871	2

Condensed pipeline results for the incense cone, agarwood chips and the *Dendrobium* stem IonTorrent clusters. Only the clusters with CITES hits are listed, for each CITES hit the cluster with the lowest e-value and highest sequence similarity percentage was selected. For simplicity’s sake columns with BLAST hit metadata (i.e. bit-score, e-value, accession numbers etc.) have been omitted, the full results are accessible in Additional file 1.

### Usage

In its simplest form, the pipeline is run on an input FASTA file, matches the BLAST results against a local copy of the CITES database, and writes the BLAST results and CITES information to an output TSV file. From the command-line interface this can be achieved with the following command:

With the default settings the sequences are matched against the NCBI GenBank nr database using a blastn search. BLAST hits are filtered with the aforementioned criteria: hits must have a maximum e-value of 0.05, a minimum hit length of a 100 bp and a minimum hit identity of 97%. The 10 hits with the lowest e-value are returned. These default settings can be altered if needed, use the argument for more details.

In addition, a user-specified blacklist file (which is a CSV file containing spuriously annotated GenBank accession numbers; Table 3) can be specified like:

Additional taxon ID databases, such as a database of additionally banned taxa, can be specified by providing the argument with multiple values.

To force or avoid updating the local CITES database, add or, respectively. Use the argument for a full list of available arguments.

Besides running the pipeline from the command-line interface it is also possible to make it available in more user-friendly environments: the pipeline can be installed as a standalone Common Gateway Interface (CGI) web application or be installed onto the galaxy platform [22,23] (necessary scripts and configuration files are provided with the distribution). In both cases the functionality of the pipeline is then available to end-users by interacting with a simple, graphical user interface.

## Results and discussion

### Caveats

The reliability and accuracy of using BLAST as a method for identification depends on several factors. Firstly, the completeness of the reference database is of importance. Very few entire genomes of CITES-listed species have been sequenced: so far only 130 [24] out of a total of 30713 species. Our pipeline is therefore not intended to handle Whole Genome Shotgun (WGS) data.

Secondly, for the standard DNA barcoding markers not all CITES-listed taxa have so far been sequenced. Species in diverse groups such as Orchidaceae or Primates are sometimes similar, and differences between their standard barcodes may therefore be small. To prevent both type I and type II errors in the identification of difficult to distinguish species, specialists of various CITES committees decided that for species that cannot be discriminated based on DNA barcodes the entire genus (that can be recognized by DNA barcoding) rather than the individual species (that cannot) should be placed on the CITES Appendices. The CITES organization annually updates the contents of its appendices for this reason.

An example case is *Cyclemys* spp., a genus of freshwater turtles (Geoemydidae): one widespread species, *C. dentata*, is heavily exploited for food while other species in the genus are rarely traded. The entire genus was placed on appendix II in 2013. In the criteria for amendment of the appendices [25] it is explicitly stated that this action was carried out because enforcement officers are unlikely to be able to distinguish traded material of *C. dentata* from close relatives (look-alike criteria set out in Annex 2b). In response to this, the default settings of the HTS pipeline use a cut-off value of 3% sequence similarity to distinguish species from each other by DNA barcodes obtained. This approach generally works to keep endangered and non-CITES protected close relatives apart from each other. We explicitly state the cases to which this does not apply below. A cut-off value was chosen based on earlier studies that found this divergence to be sufficient to keep the majority of plants and animals apart using the standard *matK*, *rbcL* and *COI* DNA barcoding markers [19,20].

Thirdly, the quality of identification depends on the length of the DNA barcode sequence used for identification. Smaller fragments have been shown to lack the discriminatory power to distinguish between species in a genus or higher taxon [18]. For this reason, the pipeline discards identifications obtained from matches shorter than 100bp by default. Finally, to minimize the chance that identifications are based on an erroneous entry the user should look, where possible, at multiple BLAST results and verify that they are in agreement with each other. The pipeline by default returns the 10 BLAST hits with the lowest e-value (after BLAST filtering); based on multiple identifications per sequence the end-user should validate whether an identification is reliable. We recommend that users select BLAST hits with the highest sequence similarity and match length wherever possible. If multiple hits are obtained with identical quality results, but different assigned species, the fragment lacks the discriminatory power to describe the hit to species level. In these cases the user should refrain from assigning a single species but stick to the genus instead.

In our experience, virtually no situations have yet occurred in which a non-CITES-protected species could be mixed up with a CITES-protected taxon. The only exceptions concern taxonomic groups that contain domesticated species from Bovidae (wild cattle, goats and sheep) and Canidae (wolves and foxes). The wild species in these taxonomic groups cannot always be distinguished from their domesticated relatives (cows, dogs, domestic goats and sheep) so identification using standard barcoding markers fails. Similar issues arise when trying to determine whether a species is cultivated or not, as standard barcodes do not provide the necessary resolution to distinguish cultivars from samples collected in the wild.

### Performance evaluation

The *HTS barcode checker* pipeline is the first tool for automated searches for DNA barcodes of CITES-protected taxa in HTS data. On the CITES website, several other online tools are available, such as databases that can be queried for information about trade, management systems, export quota, publications, identification manuals and photographs, but none as yet to search for hits in HTS datasets. The Chinese Academy of Medical Sciences in Beijing produces DNA barcodes from ingredients from Traditional Chinese Medicines and lists these on its website, but here too automatic search tools are not provided.

To compare speed of the pipeline to current practices we presented a spreadsheet file with ten taxonomic names (among which two CITES-listed taxa) obtained from a TCM HTS dataset to ten colleagues and let them search for CITES-listed taxa by scrolling through the CITES Appendices using the 'search and find’ option in Adobe Reader. Processing time ranged between little over one minute to slightly under five minutes among the ten participants and did not result in full recovery of CITES-listed taxa in all cases. The *HTS barcode checker* pipeline processed the same dataset in less than ten seconds and successfully retrieved all protected species.

### Test cases

Here we report the pipeline results for three sequence sets that were based on material confiscated by Dutch customs officials. For each sample the Internal Transcribed Spacer 1 (nrITS1) region was amplified and sequenced using the IonTorrent PCM platform. The reads were clustered using CD-HIT [16] at 97% sequence similarity. The clusters were identified with the *HTS barcode checker* pipeline under default settings (max e-value of 0.05, minimum of 97% sequence similarity and a hit length of at least a 100 bp). The full pipeline results are available in Additional file 1. The clustered FASTA files for all cases are available with the pipeline distribution in the /data folder.

Case 1

An incense cone was sequenced and clustered of which the manufacturer provided us with all ingredients among which a protected taxon (*Aquilaria*). Clustering produced a total of 175 non-singleton OCTUs. A total of 99 unique identifications could be obtained by BLASTing using the pipeline. The results, listed in Table 3, indicate that the cone indeed contained species of *Aquilaria* (Thymeleaeceae), which are all placed on CITES Appendix II. The not protected plant species specified by the manufacturer were identified as well, thus validating the method.

Case 2

Wood chips from a confiscated agarwood sample were sequenced. Clustering resulted in a total of 51 non-singleton OCTUs. A total of 26 unique identifications could be obtained by BLASTing the OCTUs, including an identification for *Aquilaria* species which is listed on CITES appendix II. The majority of the other OCTU identifications were from *Citrullus* and *Pseudomonas*.

Case 3

A confiscated *Dendrobium* stem was sequenced and clustered, this produced a total of 3845 non-singleton OCTUs. A total of 159 unique identifications could be obtained by BLASTing using the pipeline; these included three different *Dendrobium* species, listed in Table 3. The results indicate that the stem indeed belongs to a member of the *Dendrobium* genus, though the barcode lacks the discriminatory strength to determine the exact species. Since all Orchidaceae are on CITES appendix II the sample was lawfully confiscated. Other sequence results included various fungal species.

### Future directions

Although the pipeline presented here is ready to use, several enhancements are possible that would increase usability and impact. For example, although incorrect taxonomic identifications of NCBI GenBank records have previously been noted, no community project exists to record and track such errors [26]. The blacklist used by the *HTS barcode checker* could be used for communal record keeping, especially as our usage of git as a decentralized revision control system provides the ideal infrastructure for this. Conversely, should an alternative community-wide blacklist of NCBI GenBank come into existence, *HTS barcode checker* could be modified to make use of it. We expect the number of users to grow once the *HTS barcode checker* project is linked from the CITES Virtual College [27], which would build a community that could 'crowd source’ such a blacklist.

Though the *HTS barcode checker* can be setup to run via CGI or platforms such as galaxy, a publicly hosted web service would make the pipeline accessible to non-expert users such as customs officers as it would remove the need for local installations. In addition, this web application could be configured to update the local databases of additional names and the blacklist at frequent intervals, thereby guaranteeing that the user always operates on state-of-the-art knowledge.

Lastly, DNA barcodes of CITES-protected species collected from well-identified specimens should be uploaded in larger numbers to BoLD, where taxonomic names can be updated as needed by third parties. The number of CITES-protected species is currently 820 for mammals, 605 for birds, 722 for reptiles, 81 for amphibians, 20 for sharks, 132 for fishes, 3 for lungfishes, 1 for sea cucumbers, 25 for scorpions and spiders, 69 for insects, 2 for leeches, 37 for clams and mussels, 10 for snails and conches, 1636 for corals and sea anemones, 260 for sea ferns, fire corals and stinging medusae, and 26290 for plants (counts based on [28-37] and the latest proposed changes to the CITES Appendices). From this total of 30713 CITES-protected species, roughly 16830 (55%) are present in NCBI GenBank with DNA barcodes, and 13883 (45%) remain to be sequenced. Multiple initiatives carried out at The Field Museum and Missouri Botanical Garden (USA), Naturalis Biodiversity Center (the Netherlands), Muséum National d’Histoire Naturelle (France), Smithsonian’s National Museum of Natural History (USA), Zoological Institute of the Russian Academy of Sciences (Russia) and University of Johannesburg (South Africa) are currently producing additional barcode sequences of CITES-listed species. We therefore expect that the current number of 45% not yet covered in NCBI GenBank or BoLD will decrease.

## Conclusions

High-throughput sequencing of DNA barcodes has improved identification potential of traded endangered species. Taxonomic errors in reference databases such as NCBI GenBank, and incongruences in the taxonomies of the CITES appendices and DNA barcode reference databases, can lead to incorrect conclusions on illegal trade. The *HTS barcode checker* pipeline is developed for automated identification from mixtures of illegally traded species, and includes functionality for correcting and standardizing taxonomic names to overcome the caveats discussed above. The pipeline alleviates the identification process by eliminating error-prone human search and matching steps, and provides a repeatable method for assessing the presence of CITES-protected taxa by analysis of HTS data. Tests demonstrate the potential of the *HTS barcode checker* pipeline for saving manual labor, reducing taxonomic errors and increasing integration between the NCBI GenBank reference database and the CITES appendices.

## Availability and requirements

**Project name:***HTS barcode checker*

**Project home page:**https://github.com/naturalis/HTS-barcode-checker

**Operating systems:** Platform independent

**Programming language:** Python (version 2.7 or 3.0 and higher)

**Other requirements:** Python packages *biopython*, *beautiful-soup* and *requests*.

**License:** BSD-3

**Any restrictions to use by non-academics:** No

## Abbreviations

API: Application programming interface; BLAST: Basic local alignment search tool; BoLD: Barcode of life database; CITES: Convention on international trade in endangered species of wild fauna and flora; CSV: Comma separated values; HTS: High throughput sequencing; NCBI: National Center for Biotechnology Information; OCTU: Operational clustered taxonomic unit; TCM: Traditional Chinese medicine; TNRS: Taxonomic name reconciliation service; TSV: Tab separated values.

## Competing interests

The authors declare that they have no competing interests.

## Authors’ contributions

YL re-implemented a first prototype of *HTS barcode checker*. YL, RAV and BG contributed equally to the drafting of this manuscript. RAV oversaw software engineering, TP provided confiscated TCM samples for sequencing. All authors have reviewed and approved the final version of this manuscript.

## Supplementary Material

Additional file 1 **Output format for pipeline results.** This file includes the full results of the IonTorrent clusters obtained from the three test cases analyzed. Click here for file
